# Prevalence and Associated Risk Factors of *Haemonchus contortus* in Sheep Slaughtered at Bahir Dar Municipal Abattoir, Ethiopia

**DOI:** 10.1155/2024/1433264

**Published:** 2024-05-15

**Authors:** Dereje Regassa, Aschalech Birhane, Yihenew Getahun, Adugna Chalchisa

**Affiliations:** College of Veterinary Medicine, Haramaya University, PoB 138 Dire Dawa, Ethiopia

## Abstract

*Haemonchus contortus* is a blood sucking parasite resulting a massive economic loss in tropical and subtropical sheep rearing areas. A cross-sectional study was conducted to estimate the prevalence of haemonchosis and its predictors at Bahir Dar municipal abattoir, northwestern Ethiopia, from December 2022 to April 2023. A total of 378 abomasum of sheep were taken by using systematic random sampling and inspected according to standard procedures. Accordingly, the findings of this study revealed an overall prevalence of 34.40% (30/378) (95% CI: 29.75-39.35). The presence of *H. contortus* in female sheep (46.41%) was significantly higher than in male (23.35%) (*P* < 0.001). The same is true; prevalence of haemonchosis in young (<1 year) sheep was 43.33% significantly (*P* < 0.001) higher than that of adult (≥1 year) which was 26.26%. Based the origin of the animals in the current study, it was nonsignificant variation (*P* = 0.386). The current study depicted that *H. contortus* infection is a common parasitic disease and requires remarkable attention to the prevention and control of haemonchosis at the study area.

## 1. Introduction

Ethiopia has the largest livestock and draft animal population in Africa with about 70 million cattle, 42.9 million sheep, 52.5 million goats, 2.15 million horses, 10.80 million donkeys, 0.38 million mules, and about 8.1 million camels [1]. Livestock largely improves the agricultural sector of Ethiopia both as means of draft power for crop agronomy and feed income generation [2]. Small ruminants especially play a role in the economy of one country by providing extra income and support survival to poor resource farmers [3]. In Ethiopia, sheep are the second most important livestock species next to cattle and rank second in Africa and sixth in the world [4]. Sheep play an important economic role and make a significant contribution to both domestic and export markets through provision of food and nonfood (skin and wool) products. They also play a major role in the food security and social well-being of rural populations living under conditions of extreme poverty [5].

Gastrointestinal (GI) parasitic infections in livestock are a worldwide problem for both small- and large-scale farmers. Infection by GI parasites in ruminants, including sheep and goats, can result in severe economic losses in a variety of ways: reproductive inefficiency, decreased work capacity, involuntary culling, diminished food intake, poor animal growth rates and lower weight gains, treatment and management costs, and mortality in heavily parasitized animals. Among the GI parasites that cause losses in livestock, the barber's pole worm *H. contortus* is the predominant. *H. contortus* is bloodsucking, highly pathogenic, and economically important nematode that infects small ruminants [6]. Traditionally, haemonchosis was more commonly seen in known high-risk areas; however, climate change and warming of new areas of the planet appear to be allowing *H. contortus* to survive and flourish in previously low-risk zones [7].

Haemonchosis is primarily a disease of tropical and subtropical regions. However, high humidity, at least in microclimate of the feces and the herbage, is also essential for larval development and their survival. The frequency and severity of the disease largely depend on the rainfall in any particular area. Sheep and goats suffer more frequently from haemonchosis [8].

The major impact of *H. contortus* in small ruminants is associated with the bloodsucking activity of the parasites which is responsible for extensive loss of blood, with each worm sucking 0.05 milliliter of blood per a day [9].

Haemonchosis, if untreated, can lead to protein deficiency, anemia, bottle jaw, swelling of the lower jaw as a result of anemia, general digestive disturbances, and death [10]. Diagnosis is made on the basis of clinical signs, grazing history season; demonstrating eggs of fecal examination is confirmative, but specific identification is difficult and required specialized laboratories and observation of adult parasite during postmortem examination [11].

*H. contortus* causes retarded growth, low productivity, and poor reproductive performance which can lead to loss of meat 27% and wool 40% among sheep/goats [12, 13]. *H. contortus*, as the highest egg producer of all sheep worms, is one of the more devastating internal parasites [14]. Comprehensive evidence is essential to develop effective prevention and control plans to mitigate the parasite. Even though a lot of research has been done in different areas, there is no recent research done in the study area. Therefore, the objectives of this study were to estimate the recent prevalence of haemonchosis and related risk factors in sheep slaughtered at Bahir Dar municipal abattoir.

## 2. Materials and Methods

### 2.1. Study Area

**Figure 1:**
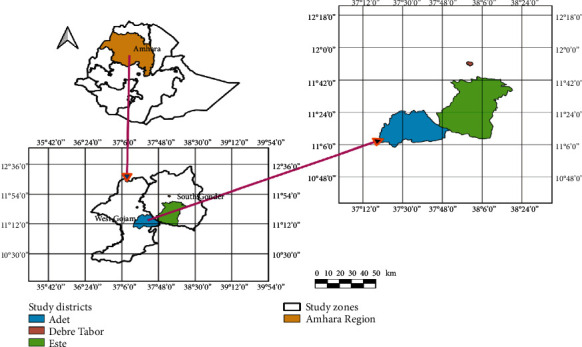
Study area map.

The study was conducted at Bahir Dar municipal abattoir, which is located at northwestern part of Ethiopia, 565 km from Addis Ababa. Bahir Dar is the capital city of Amhara regional state and situated in the southwest direction of Lake Tana. The altitude of the area is 1810 to 1850 meters above sea level. Bahir Dar is located between 12° 29′ N latitude and 37° 29′ E longitude. The town receives an average annual rain fall ranging from 1200 mm to 1600 mm, with annual temperature ranging from 8°C to 31°C (National Meteorological Agency, Bahir Dar, Ethiopia, 2019). The study animals were bought from three woredas: Adet is located south of Bahir Dar in the Mirab Gojjam Zone of the Amhara Region, and this town has a latitude and longitude of 11° 16′ N and 37° 29′ E with an altitude of 2216 meters above sea level; Este was one of the 105 woredas in the Amhara Region of Ethiopia. As part of the Debub Gondar Zone, Este was bordered on the south by the Abay River which separated it from the Misraq Gojjam Zone, on the west by Dera, on the northwest by Fogera, on the north by Farta, on the northeast by Lay Gayint, and on the east by Simada, and this town has a latitude and longitude of 11° 39′ 59.99^″^ N and 38° 09′ 60.00^″^ E with an altitude, and Debre Tabor is a town and woreda in northern Ethiopia. Located in the Debub Gondar Zone of the Amhara Region, about 100 kilometers southeast of Gondar and 50 kilometers east of Lake Tana, this historic town has a latitude and longitude of 11° 51′ N and 38° 1′ E with an elevation of 2706 meters above sea level (Figure 1).

### 2.2. Study Animal

The study animals were sheep slaughtered in Bahir Dar municipal abattoir. The sheep were indigenous breeds kept under traditional management system. The study animals were of different sex, age, origin, and body condition. In this study, the origins of the animals were recorded from merchants who brought animals to the abattoir. The age of the sheep was characterized using teeth eruption. The body condition score was determined according to [15] as poor, medium, and good.

### 2.3. Study Design

A cross-sectional study using systematic random sampling technique was conducted from December 2022 to March 2023 to determine the prevalence of haemonchosis in sheep slaughtered at Bahir Dar municipal abattoir.

### 2.4. Sample Size Determination

The sample size was determined by using the standard formula described in [16] by considering an expected prevalence of 56.25% done in [17] at the study area with precision of 5% and 95% confidence level.

(1) $$\begin{array}{c} \frac{n=z^{2}p_{\exp}\;\left(1-P_{\exp}\right)}{d^{2}}, \end{array}$$

where *n* is the minimum required sample size for the study, *d* is the absolute desired precision, and *P*_exp_ is the expected prevalence in the study area. Therefore, the minimum required sample size was 378 animals.

### 2.5. Study Methodology

A few hours before slaughter, antemortem investigation was performed and properly recorded for each animal concerning its origin, sex, age, breed, and its general health condition. The animals were selected by systematic random sampling, and the abomasa were removed from the abdominal cavity and opened along their greater curvature. Close visualization was made for the presence of adult *H. contortus*. The worms were collected in normal saline and identified based on the characteristics [9].

### 2.6. Data Analysis and Management

The data recorded from both antemortem and postmortem inspection was cleaned, edited, verified, and stored in Microsoft Excel spreadsheet program version 13 and finally imported into Stata software program version 16 for analysis. Both descriptive statistics (proportion and standard deviation) and inferential statistics (chi-square test and binary logistic regression) were performed. Univariable and multivariable binary logistic regressions were used to analyze the association of exposure variables with *H. contortus*. In all cases, 95% of confidence intervals and *P* < 0.05 were considered as statistically significant. The goodness of fit test for the logistic regression model was confirmed by using the Hosmer-Lemeshow test. The model was fitted to the sheep dataset with *χ*^2^ = 9.20, df = 8, and *P* value = 0.3257.

## 3. Results

**Figure 2:**
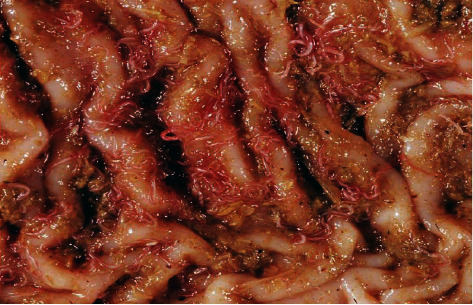
*H. contortus* in abomasum.

**Figure 3:**
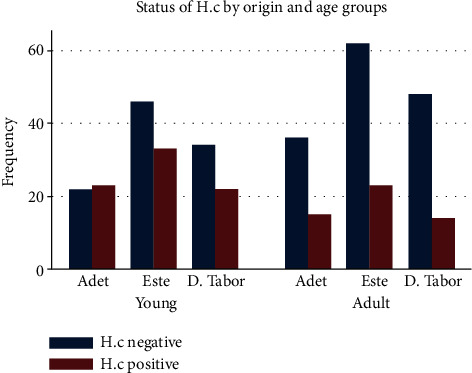
Status of *H. contortus* by age and origin.

This study revealed that out of 378 inspected sheep, 130 (34.40%) (95% CI: 29.75-39.35) were infected with haemonchosis (Table 1 and Figure 2). From the 197 male and 181 female sheep examined, 46 (23.35%) and 84 (43.41%) were positive for *H. contortus*, respectively (Table 1). Based on origin of sampled sheep, 96, 164, and 118 were brought from Adet, Este, and D. Tabor, respectively. From these, 38 (39.58%), 56 (31.15%), and 36 (30.54%) were positive for *H. contortus*, respectively (Table 1). Based on age, 180 young and 198 adult sheep were examined and 78 (43.33%) and 52 (26.26%) were positive, respectively (Table 1 and Figure 3).

The prevalence of *H. contortus* was also higher in adult sheep originated from Este followed by adult originated from Adet (Figure 3).

By using chi-square test, the variables age and sex had statistical significant association (*P* value < 0.05) with the prevalence *H. contortus* in sheep whereas origin had not (*P* value > 0.05) (Table 1).

In this study, the prevalence of haemonchosis in young (<1 year) sheep was 43.33% (Table 1).

### 3.1. Factors Associated with the Prevalence of Haemonchosis

Variable screening was conducted by using bivariate (crude) analysis of each independent variables with prevalence of haemonchosis at 5% level of significance. The crude variable analysis revealed that two variables (age and sex) showed statistically significant association with haemonchosis. On the other hand, the covariate origin was not significantly associated with haemonchosis. Even though the origin had no significant association in the bivariate analysis, it was incorporated in the multivariable analysis due to limitation of factors and its important nature of confounding effect. Therefore, four covariates were included in the multivariable analysis.

In the multivariable logistic regression model, the CI of AOR for two covariates (female sex and young age) does not include 1 (*P* value < 0.05) at the 5% level of significance. This indicates that these factors were statistically significant predictor of the prevalence of *H. contortus*. For female sheep, the odds of developing *H. contortus* increases by 2.66 fold compared to males by holding other variables in the model constant (AOR = 2.66, 95% CI = 1.67-4.23, *P* < 0.001). Young sheep also had a statistically significant association with prevalence of *H. contortus*. Being an adult sheep reduces the odds of developing *H. contortus* by 57% compared to young animals by adjusting for other variables (AOR = 0.43, 95% CI = 0.27-0.68, *P* < 0.001). However, the prevalence of *H. contortus* did not show statistically significant difference between the different origins of sheep, since the CI for the AOR for this covariate includes 1 (*P* value > 0.05) (Table 2). The goodness of fit test for the logistic regression model was confirmed by using the Hosmer-Lemeshow test. The model was fitted to the sheep dataset with *χ*^2^ = 9.20, df = 8, and *P* value = 0.3257.

## 4. Discussion

Haemonchosis is a serious health problem that leads to a decline in production due to high morbidity, mortality, cost of treatment, and control measures [18].

The present study revealed that the overall prevalence of *Haemonchus contortus* in sheep was 34.40%, which indicated high prevalence of the parasite in the study area. This finding was much lower compared the report in different areas of Ethiopia: 81.35% in Kombolcha [19], 69.6% in Bishoftu [9], 69.5% in Bedelle town [20], 91.2% in Bishoftu [21], 96.5% in arid and semiarid zones of eastern Ethiopia [22], 80.21% in Gondar town [23], 90.1% in Haramaya [24], 61.63% in Wolaita Sodo town [25], and 63.68% in Bishoftu [26].

The differences in the prevalence of haemonchosis in sheep in various parts of Ethiopia and other countries could be attributed to factors like sample size and environmental conditions (humidity, temperature, and rainfall). On other hand, host factor, level of education, economic status of the community, and awareness among community about proper management practices and anthelmintic use could also contribute to variations in prevalence across different areas.

In the present study, a higher prevalence of haemonchosis was observed in female sheep compared to males with a statistically significant difference between the sexes (Table 2). The present prevalence of haemonchosis in male and female sheep varies from previous findings. Previous studies reported rates of 80.9% and 77.1% in males and females, respectively, in Gondar town [23]; 73.22% and 64.71% in males and females, respectively, in and around Finote Selam in Amhara Region [27]; and 66.0% and 59.7% in males and females, respectively, in Arsi Negele in Oromia region [28]. In all reports, male sheep were more infested by Haemonchus than female sheep. But some report revealed that female animals are more susceptible than male animals due to cyclic hormonal variation [17]. It is assumed that sex is a determinant factor influencing prevalence of haemonchosis, with females being more susceptible to parasitism due to reproductive stress and decreased immune status [9] which supports the current finding.

The current study found a higher prevalence of haemonchosis in young animals (43.33%) compared to adults (26.26%). There was a statistically significant difference between the two age groups. This is in line with previous findings that young animals had a higher prevalence of 66.9% compared to the 59.0% prevalence in adults in Debre Zeit Elfora export abattoir in Bishoftu town [9]. The higher infection rate in young animals might be due to their greater susceptibility to the parasite. This also explained that young sheep have less or no previous exposure to the parasite compared to adults. Young sheep, during their first year of life, primarily feed and graze on grasslands, which expose them to parasites. Conversely, the lower parasitism in the adult animals is due to the development of significant immunity with the course of time. Gradually, as the exposure to parasitic infection increases, the immune system of host animals especially against *Haemonchus* species builds up through age resistance.

## 5. Conclusion

The current study depicted that *Haemonchus contortus* infection is a common parasitic disease and requires remarkable attention to its prevention and control in the study area, with an observed prevalence of 34.40%. The prevalence of haemonchosis in this study area was statistically significantly with sex and age groups. However, the origin was not statistically significantly associated with prevalence of haemonchosis. Special attention should be paid to newborn and young sheep, purchase of male sheep rather than females for slaughter at abattoirs, and the application of anthelmintic treatment on animals brought from different areas prior to their slaughter.

## Figures and Tables

**Table 1:** The overall prevalence and the association of predictors of haemonchosis in sheep at Bahir Dar municipal abattoir.

Predictors	Categories	Number examined (%)	Number positive (%)	df	*P* value
Sex	Male	197 (52.12)	46 (23.35)	1	<0.001^∗^
	Female	181 (47.88)	84 (46.41)		

Age	Young	180 (47.62)	78 (43.33)	1	<0.001^∗^
	Adult	198 (52.38)	52 (26.26)		

Origin	Adet	96 (25.40)	38 (39.58)	2	0.386
	Este	164 (43.39)	56 (34.15)		
	D. Tabor	118 (31.22)	36 (30.54)		

*χ* ^2^: chi-square value; df: degree of freedom. ^∗^Statistical significance.

**Table 2:** Analysis of *H. contortus* prevalence in sheep with different potential factors using univariable and multivariable binary logistic regression analyses.

Predictors	Categories	COR (95% CI)	COR (95% CI)	*P* value
Sex	Male	Ref	Ref	<0.001^∗^
	Female	2.84 (1.83-4.42)	2.66 (1.67-4.23)	

Age	Young	Ref	Ref	<0.001^∗^
	Adult	0.47 (0.30-0.72)	0.43 (0.27-0.68)	

Origin	Adet	Ref	Ref	
	Este	0.79 (0.47-1.33)	0.74 (0.42-1.29)	0.284
	D. Tabor	0.67 (0.38-1.18)	0.71 (0.39-1.31)	0.273

CI: confidence interval; COR: crude odds ratio; AOR: adjusted odds ratio; A*β*: adjusted coefficients. ^∗^Statistical significance.

## Data Availability

The data is included in the manuscript.
